# Expression of Purinergic and Endothelial Activation Markers in Brain Tissue From Fatal Microcephaly Associated With ZIKV

**DOI:** 10.1002/iid3.70382

**Published:** 2026-03-11

**Authors:** Jorge Rodrigues de Sousa, Gustavo Batista Ferro, Leticia Vieira Teixeira, Carlos Silva, Ligia Lima, Orlando Neto, Raimunda Azevedo, Jannifer Oliveira Chiang, Livia Medeiros Neves Casseb, Arnaldo Jorge Martins Filho, Lívia Caricio Martins, Juarez Antônio Simões Quaresma, Pedro Fernando da Costa Vasconcelos

**Affiliations:** ^1^ Departamento de Patologia Universidade do Estado do Pará Belém Pará Brazil; ^2^ Faculdade de Medicina Universidade do Estado do Pará Belém Pará Brazil; ^3^ Instituto de Patologia Cirúrgica e Molecular Belém Pará Brazil; ^4^ Seção de Patologia Instituto Evandro Chagas Ananindeua Pará Brazil; ^5^ Seção de Arbovirologia e Febres Hemorrágicas Instituto Evandro Chagas Ananindeua Pará Brazil

**Keywords:** microcephaly, neuroinflammation, purinoceptors, Zika virus

## Abstract

**Background:**

Zika virus (ZIKV) is a flavivirus that has gained global attention due to its association with congenital microcephaly and neuroinflammatory responses. Markers of endothelial activation and purinergic signaling have been identified in the context of ZIKV neuropathogenesis, although the underlying mechanisms remain poorly understood.

**Methods:**

Brain tissue samples from fatal cases of ZIKV‐induced microcephaly were analyzed using immunohistochemistry to detect endothelial activation markers (E‐selectin, P‐selectin, ICAM‐1, and VCAM‐1) and purinergic receptors (P2X4, P2X7, and P2Y2). Quantitative analysis measured the expression patterns of these molecules and assessed their contribution to neuroinflammation and blood–brain barrier disruption.

**Results:**

ZIKV‐positive cases exhibited significant endothelial activation, with increased expression of adhesion molecules mediating leukocyte recruitment. Purinergic receptor upregulation suggested a role in excitotoxicity and neuroinflammatory exacerbation. Statistical analysis revealed a marked difference in marker expression between ZIKV‐infected cases and controls (*p* < 0.0001).

**Conclusion:**

The interaction between endothelial activation and purinergic signaling may be associated with the vascular dysfunction and neuronal damage observed in ZIKV‐associated microcephaly. Understanding these associations could contribute to the development of targeted interventions for Zika congenital syndrome.

## 1 Introduction

ZIKV is a non‐segmented, enveloped, positive‐sense RNA virus belonging to the family Flaviviridae, genus Orthoflavivirus. Its genome consists of a single open reading frame encoding a polyprotein, which is cleaved into 10 viral proteins: three structural proteins—envelope (E), membrane (M), and capsid (C)—and seven non‐structural proteins (NS1, NS2A, NS2B, NS3, NS4A, NS4B, and NS5) [1]. These proteins are essential for modulating host immune responses, as well as for viral replication, processing, and assembly [2].

First isolated in 1947 from a sentinel rhesus monkey in Uganda, ZIKV remained relatively obscure until the 2015 outbreak in the Americas. This epidemic raised global concern due to its causal association with Guillain‐Barré syndrome and a dramatic surge in cases of congenital microcephaly among newborns of women infected during the first trimester of pregnancy [3].

From an immunopathogenic perspective, the host immune response is critical for shaping the course of ZIKV infection. The balance between pro‐ and anti‐inflammatory signals determines not only viral clearance but also the extent of tissue damage, particularly through immune evasion strategies employed by the virus [4]. These mechanisms often target immune‐privileged organs such as the placenta, testes, and brain—sites characterized by tightly regulated blood‐tissue barriers. Within these compartments, endothelial cells are central to maintaining immune surveillance and homeostasis [5].

In the context of the blood–brain barrier (BBB), one of the major challenges in understanding ZIKV neuropathogenesis lies in elucidating how the virus infects endothelial cells and compromises barrier integrity. ZIKV‐induced alterations in endothelial permeability facilitate immune cell infiltration and contribute to neuroinflammation [6]. The disruption of homeostasis and synaptic signaling—driven by interactions among astrocytes, neurons, and microglia—creates a cytotoxic microenvironment [7]. This environment may lead to the activation of purinergic receptors (P2X4, P2X7, and P2Y2), which are implicated in neuronal damage and excytotoxicity. These receptors are also associated with hypoxia‐induced endothelial dysfunction observed in severe ZIKV‐associated microcephaly [8].

Purinergic receptors constitute a family of receptors activated by extracellular nucleotides, primarily ATP, which play essential roles in cell signaling within the central nervous system (CNS). These receptors are categorized into two main groups: P1 receptors, which are sensitive to adenosine, and P2 receptors, which respond to nucleotides like ATP and ADP. P2 receptors are further subdivided into ionotropic (P2X) and metabotropic (P2Y) subtypes, each with distinct signal transduction mechanisms and pathophysiological functions [8].

In the context of neuroinflammation, purinergic receptors have been extensively studied due to their role in modulating glial activation and the release of pro‐inflammatory mediators. The P2X7 receptor, in particular, is prominent for its ability to form a membrane pore in response to high levels of extracellular ATP, a condition that occurs during neural injury and chronic inflammatory states. P2X7 activation promotes the release of inflammatory cytokines such as IL‐1β, in addition to inducing apoptosis and necrosis, thereby contributing to the amplification of neural damage [6].

In addition to P2X7, the metabotropic P2Y receptors, especially P2Y12, play a crucial role in modulating microglia, the CNS's resident immune cells. P2Y12 activation regulates microglial migration and the release of factors that influence the cerebral inflammatory microenvironment. This regulation is fundamental for maintaining the balance between neuroprotective and neurotoxic processes during neuroinflammation [7].

Here, our study demonstrated in situ activation of purinergic receptors and endothelial expression of adhesion molecules—including E‐selectin, P‐selectin, ICAM‐1, and VCAM‐1—in fatal cases of congenital ZIKV infection. Thus, the present study explored how these pathways contribute to leukocyte recruitment, rolling, and transmigration, ultimately promoting the formation of inflammatory infiltrates and the histopathological features of severe microcephaly.

## 2 Methods

### 2.1 Characterization of the Sample

Brain tissue samples were collected from nine patients. Six of these were fatal cases of ZIKV‐induced microcephaly, confirmed by positive RT‐qPCR and/or viral isolation in cell culture or immunohistochemistry. The remaining three cases constituted a negative control group, who died from other causes and showed preserved neural architecture. For histopathological analysis, 5 µm sections were obtained from paraffin‐embedded tissue samples and stained with hematoxylin and eosin.

### 2.2 Immunohistochemistry

The immunohistochemical procedure for tissue immunolabeling with marker‐specific antibodies was based on the formation of a biotin–streptavidin–peroxidase complex. The protocol was optimized for the detection of ZIKV antigens using a polyclonal anti‐ZIKV antibody produced at the IEC, which recognizes both structural and non‐structural viral epitopes. In this context, antibody validation and antigen detection were performed using a conjugation‐based system that does not require avidin or biotin. Protocol adaptations were carried out in accordance with previously published methodologies [4, 5]. Tissue deparaffinization and hydration were performed using xylene followed by a series of ethyl alcohol baths at concentrations of 90%, 80%, and 70%. Endogenous peroxidase was blocked with 3% concentrated hydrogen peroxide (H_2_O_2_) in three 15‐min incubations. Antigen retrieval was then performed with citrate buffer (pH 6.0) for 20 min at 90°C.

Nonspecific proteins were blocked with 10% concentrated skim milk for 30 min. After this step, the histological sections were incubated with primary antibodies diluted in 1% Bovine Serum Albumin (BSA) for 14 h (Table 1). After this interval, the slides were immersed in a 1× PBS‐buffer solution and then incubated with the biotinylated secondary antibody LSAB (DakoCytomation) for 30 min at 37°C. Following the first incubation, the slides were again immersed in 1× PBS and incubated with streptavidin peroxidase (LSAB DakoCytomation) for 30 min at 37°C.

**Table 1:** Primary antibodies used for endothelial and purinoreceptor phenotype in fatal cases of ZIKV‐associated microcephaly.

Antibody	Reference	Dilution
E‐selectin	Novus Biologicals (NBP1‐45545)	1:100
P‐Selectin	Novus Biologicals (NBP2‐22046)	1:100
VCAM‐1	Abcam (ab134047)	1:100
ICAM‐1	Novus Biologicals (NBP2‐22540)	1:100
P2Y2	Abcam (NB110‐3932)	1:100
P2X4	Abcam (ab134559)	1:100
P2X7	Novus Biologicals (NBP1‐20140)	1:100

*Note:* Primary antibodies used for the immunohistochemical detection of endothelial adhesion molecules and purinergic receptors in brain tissue from fatal cases of Zika virus‐associated microcephaly. The table includes the antibody target, supplier reference, and working dilution applied for each marker.

Finally, the sections were developed by applying a chromogen solution composed of 0.03% diaminobenzidine and 3% hydrogen peroxide. At the end of the developing stage, the preparations were washed in distilled water and then counterstained with Harris hematoxylin for 1 min. The histological sections were then dehydrated in increasing concentrations of ethyl alcohol and finally cleared in xylene.

### 2.3 Quantitative Analysis and Photodocumentation

Cell counts were performed using the Zen Blue software (Zeiss), through manual analysis of images obtained by light microscopy at 400× magnification. All images were standardized in terms of lighting and contrast to ensure consistency across the analyzed fields.

The counting was conducted in a blinded manner, ensuring that the observers did not have access to the identification of the experimental groups during the analysis. Two independent observers, previously trained to recognize the morphological criteria established in the study, participated in the process. Differences exceeding 10% between counts were resolved by joint re‐evaluation until a consensus was reached.

Photodocumentation was also performed with the Zen Blue software, with the selection of representative images based on the quality of focus, contrast, and integrity of the cellular structures.

Sample analyses were conducted using an AXIO IMAGER Z1‐ZEISS microscope (model 4560006). The quantification of the immunolabeling was performed by randomly selecting 10 high‐magnification fields representative of the neural parenchyma. Each field was subdivided into 10 × 10 quadrant areas, delineated by a reticle, totaling an area of 0.0625 mm² per field.

### 2.4 Statistical Analysis

The results obtained from the experiments were stored in electronic spreadsheets. Statistical analysis was performed using GraphPad Prism 5.0 software. In the univariate analysis, frequencies, measures of central tendency, and dispersion were obtained. For the investigation of the hypotheses, the Student's *t*‐test and Pearson correlation were applied. All tests were conducted considering a significance level of 5% (*p* ≤ 0.05).

## 3 Results

### 3.1 Histological Description of the Main Tissue Changes and Immunoexpression of Endothelial Macardors and Purinergic Receptors in Fatal Cases of ZIKV‐Induced Microcephaly

**Figure 1:**
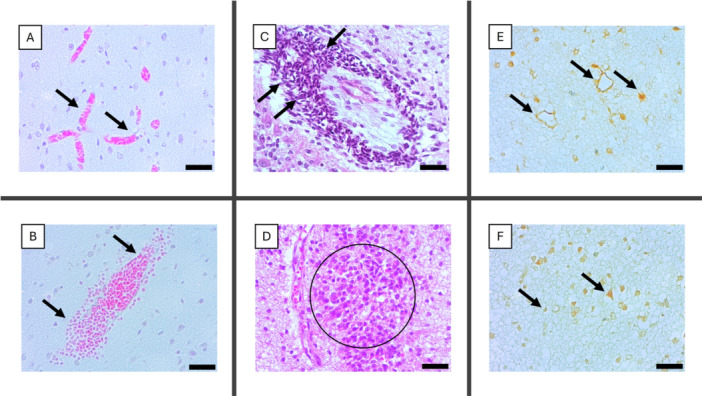
Histopathological and immunohistochemical findings in fatal cases of microcephaly associated with ZIKV infection. (A) Vascular congestion in the brain parenchyma is evidenced by dilated blood capillaries filled with red blood cells (black arrows). (B) Focal hemorrhage in the nervous tissue with extravasation of red blood cells into the extracellular space (black arrows). (C) Perivascular inflammatory infiltrate surrounding small vessels (black arrows). (D) Presence of a glial nodule characterized by astrocytes and activated microglia (black circle). (E) Positive immunostaining for anti‐ZIKV antibody in vascular endothelium (black arrows) and in cells with morphology consistent with astrocytes (red arrows). (F) ZIKV positivity was also observed in cells with morphology consistent with microglia (black arrows) and neurons (red arrows). Scale bar, 50 μm (400× magnification).

In fatal cases of ZIKV‐positive congenital microcephaly, histopathological examination revealed significant alterations in the intraparenchymal environment. The white matter exhibited marked edema, and the cerebral cortex showed a proliferation of capillaries and endothelial cells with evident vascular congestion (Figure 1A). Histological analysis also revealed focal hemorrhagic areas (Figure 1B). A prominent feature in the parenchyma was the extensive presence of perivascular inflammatory infiltrates (Figure 1C) and glial nodules (Figure 1D).

Immunohistochemistry using an anti‐ZIKV antibody revealed positivity in various cell types within the central nervous system of fatal cases of congenital microcephaly. Endothelial immunolabeling appeared as a brown‐orange cytoplasmic staining, indicating the presence of viral antigen in endothelial cells of capillaries and small‐caliber vessels (Figure 1E, black arrows). Astrocytes exhibited positive immunoreactivity primarily in their cellular processes, with a diffuse pattern observed in areas of reactive gliosis (Figure 1E, red arrows). Microglial cells showed an activated morphology and intense cytoplasmic staining, with a focal distribution in regions displaying necrosis and tissue disorganization (Figure 1F, black arrows). Infected neurons presented granular cytoplasmic immunolabeling, predominantly in the deeper cortical layers (Figure 1F, red arrows).

### 3.2 Endothelial Activation and Purinergic Dysregulation in ZIKV‐Associated Neuroinflammation and Microcephaly

**Figure 2:**
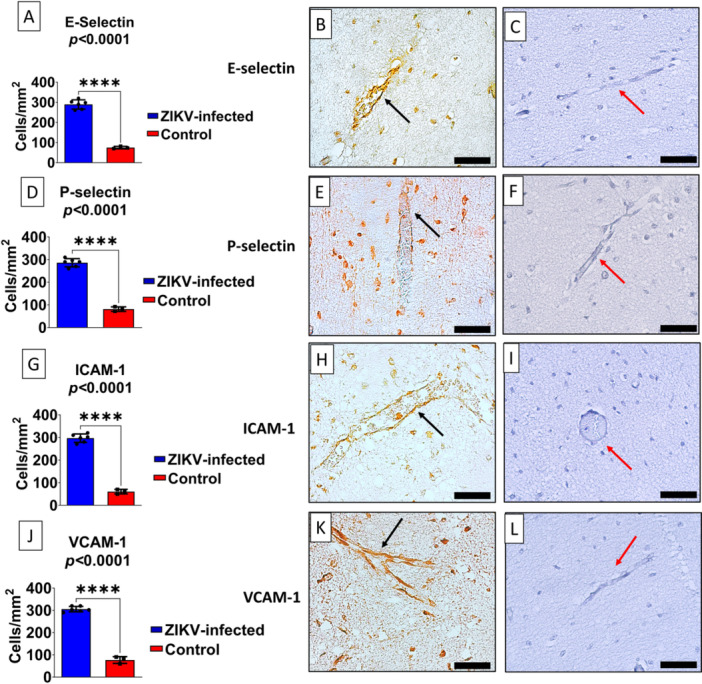
Increased Expression of Endothelial Adhesion Molecules in the Brain of Fatal Cases of ZIKV‐Associated Microcephaly. (A, D, G, J) Quantification of the density of positive cells/mm² for E‐selectin, P‐selectin, ICAM‐1, and VCAM‐1 in brain sections from cases with congenital ZIKV infection (blue bars) compared with non‐infected fetal/infant controls (red bars). All markers showed highly significant increases in ZIKV‐positive cases (****p < 0.0001). (B, E, H, K) Representative immunohistochemistry images of brain tissue from ZIKV‐infected cases, demonstrating enhanced immunoreactivity for adhesion molecules, with evident staining in vascular structures (black arrows). (C, F, I, L) Brain tissues from non‐infected controls, showing minimal or absent immunoreactivity (red arrows). Scale bar: 50 μm for all images (400× magnification).

Immunohistochemical analysis demonstrated significantly increased expression of endothelial adhesion molecules in the brains of neonates with congenital ZIKV infection compared with controls (Figure 2). A marked elevation in the density of E‐selectin, P‐selectin, ICAM‐1, and VCAM‐1 positive cells was observed, with *p* < 0.0001 for all markers. These molecules exhibited predominant distribution along the vascular endothelium, with intense membranous and cytoplasmic staining (Figure 2B,E,H,K, black arrows), consistent with widespread endothelial activation. In contrast, controls displayed weak and focal immunoreactivity, restricted to a few isolated vessels (Figure 2C,F,I,L, red arrows).

The concomitant increase in E‐ and P‐selectin supports their role in the early stages of leukocyte recruitment, facilitating rolling and the initiation of firm adhesion, whereas the upregulation of ICAM‐1 and VCAM‐1 is associated with transendothelial migration and sustained recruitment of lymphocytes and monocytes into the brain parenchyma. These findings indicate a robust inflammatory state induced by ZIKV infection, compatible with BBB dysfunction and amplification of neuroinflammation.

**Figure 3:**
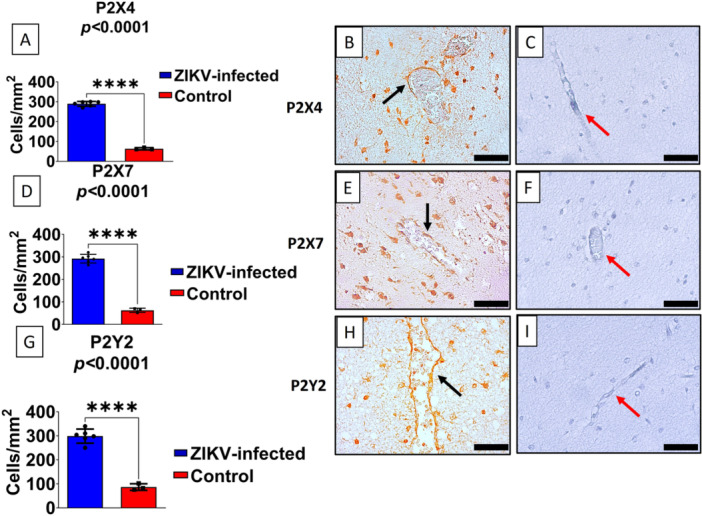
ZIKV infection increases the expression of purinergic receptors P2X4, P2X7, and P2Y2 in the human brain. (A, D, G) Quantification of the number of positive cells/mm² for the purinergic receptors P2X4 (A), P2X7 (D), and P2Y2 (G) in brain tissue samples from ZIKV‐infected individuals (blue bars) and non‐infected controls (red bars). Statistical analysis revealed a highly significant increase in receptor‐positive cell density in the ZIKV group compared with controls (****p < 0.0001). (B, E, H) Representative immunohistochemistry images showing intense staining for P2X4 (B), P2X7 (E), and P2Y2 (H) in blood vessels and adjacent cells of ZIKV‐infected brains (black arrows). (C, F, I) Corresponding control brain tissues showing markedly reduced expression of the same receptors (red arrows). Scale bar: 50 μm (400× magnification).

In addition, evaluation of purinergic receptor expression revealed significant upregulation of P2X4, P2X7, and P2Y2 subtypes in the brains of neonates with congenital ZIKV infection (Figure 3). The number of positive cells was markedly higher in infected brains compared with controls (*p* < 0.0001 for all receptors). Staining was particularly intense around blood vessels and in parenchymal cells, including astrocytes and microglia, as evidenced by perivascular localization and characteristic cellular morphology (Figure 3B,E,H, black arrows). In controls, immunoreactivity was minimal and restricted (Figure 3C,F,I, red arrows).

To quantitatively support the immunohistochemical findings, the number of positively stained cells per mm² was measured for each endothelial marker and purinergic receptor in the parenchyma of ZIKV‐positive cases and compared with uninfected controls. The results, summarized in Table 2, reveal a statistically significant increase in all evaluated markers, further confirming the pronounced endothelial activation and purinergic signaling dysregulation in fatal ZIKV‐associated microcephaly.

**Table 2:** Endothelial and purinoreceptor phenotype in neural parenchyma of fatal ZIKV‐associated microcephaly.

Markers	Parenchyma (Cells/mm^2^)	Control (Cells/mm^2^)	*p*‐value
E‐selectin	289.2 ± 22.93	75.00 ± 6.24	0.0001****
P‐Selectin	286.0 ± 18.11	81.33 ± 10.07	0.0001****
VCAM‐1	305.5 ± 12.13	76.67 ± 15.28	0.0001****
ICAM‐1	296.7 ± 16.66	60.67 ± 10.07	0.0001****
P2Y2	298.7 ± 29.36	86.67 ± 13.61	0.0001****
P2X4	289.3 ± 12.04	63.33 ± 5.77	0.0001****
P2X7	292.0 ± 19.14	62.67 ± 9.45	0.0001****

*Note:* Student's *t*‐test.

**** *p* < 0.0001.

## 4 Discussion

From the perspective of the pathogen–host relationship, the immunological mechanisms involved in the immunopathogenesis of ZIKV infection—particularly in fatal cases of microcephaly—remain incompletely understood [9]. It is known that increased vascular permeability in the neural parenchyma may result from the interplay of multiple factors, including endothelial cell phenotypes that modulate host immune responses [10].

In this context, the recruitment of immune cells in arboviral infections underscores the central role of chemokines in attracting various cell types, particularly those of lymphocytic and monocytic lineages, to sites of infection [11]. In situ studies on yellow fever virus (YFV) have demonstrated that the orchestration of the inflammatory response involves the upregulation of adhesion molecules such as E‐ and P‐selectins, which are crucial for initiating leukocyte rolling on the endothelium [12].

Notably, elevated expression of these selectins has also been observed in hemorrhagic and fatal human cases, particularly in highly vascularized organs such as the liver. In these instances, E‐ and P‐selectins were predominantly expressed in the hepatic lobule, especially in the portal tract—where inflammatory infiltrates were prominent—and in the medial zone, which showed features of coagulative and lytic necrosis [12, 13]. The infiltration of leukocytes expressing these markers suggests that cellular injury may be exacerbated by the local release of reactive oxygen and nitrogen species (ROS and RNS) [14].

In fatal cases of ZIKV‐associated neonatal microcephaly, a similar pathological pattern was observed in the neural parenchyma. The expression of endothelial adhesion molecules, including E‐selectin, P‐selectin, ICAM‐1, and VCAM‐1, was significantly increased in ZIKV‐positive samples compared with controls. In parallel, purinergic receptors (P2X4, P2X7, and P2Y2) were also upregulated in infected tissues.

Our findings are consistent with previous reports demonstrating modulation of purinergic receptors during viral infections. Notably, Chen et al. (2019) [15] showed that the P2Y2 receptor is upregulated in human cytomegalovirus (HCMV)‐infected tissues, a process associated with altered calcium signaling, enhanced viral replication, and changes in cell motility. These observations support the concept that purinergic signaling represents a conserved mechanism exploited by viruses to manipulate host cell physiology and promote infection and dissemination [15].

In the present study, immune activation markers were detected in both CNS‐resident cells (endothelial cells, astrocytes, microglia, and neurons) and infiltrating immune cells during ZIKV infection, indicating a multifaceted inflammatory response. Similarly, Qiu et al. (2025) [16] demonstrated in an immunocompromised murine model that flavivirus infection induces robust activation of resident CNS cells while promoting immune cell infiltration, thereby contributing to neuroinflammation and potential neurodegeneration.

The combined activation of resident and infiltrating cells may have important functional consequences. Activated microglia and astrocytes can exert neuroprotective effects through phagocytosis and regulatory cytokine production, but may also drive tissue damage when inflammation becomes excessive. Moreover, the expression of activation markers in neurons suggests direct viral effects or cytokine‐mediated injury, potentially leading to altered neuronal excitability and synaptic dysfunction. Together, these data reinforce the notion that the pathogenesis of neurotropic viruses such as ZIKV is driven not only by viral presence but also by host immune responses that can generate deleterious bystander effects [16].

Cell death is a hallmark of ZIKV‐induced microcephaly, particularly in fatal cases. The presence of inflammatory cells in close proximity to neurons and astrocytes exhibiting morphological signs of apoptosis and necrosis strongly suggests that immune cell infiltration contributes directly to tissue damage. In this scenario, adhesion molecules such as E‐ and P‐selectin, ICAM‐1, and VCAM‐1 are key mediators in stabilizing cell–cell interactions and facilitating leukocyte transmigration across the endothelium [17]. Their expression, localized predominantly in vascular endothelial cells and infiltrating immune cells, reflects an active and targeted immune response.

Furthermore, elevated levels of pro‐inflammatory cytokines consistent with a Th1 immune profile were observed primarily in perivascular regions and the meninges—areas associated with meningoencephalitis and immune cell infiltration [18]. Previous studies have indicated that NF‐κB signaling plays a central role in this process, driving the expression of cytokines such as TNF‐α and IL‐1β, which in turn stimulate the upregulation of ICAM‐1 and VCAM‐1 [19]. These findings reinforce the concept of endothelial activation as a critical factor in shaping the host immune response.

Nevertheless, one of the most significant challenges in understanding the pathophysiology of ZIKV infection in fatal microcephaly cases lies in unraveling the mechanisms governing BBB integrity and endothelial regulation [10].

The immune evasion strategies employed by ZIKV allow it to escape the host's innate defenses and invade immunologically privileged tissues, such as the fetal brain and eye, potentially leading to dysregulation of critical neurological processes and contributing to severe brain injuries and neurological complications associated with infection [20]. These disruptions can impair intercellular communication and may involve alterations in purinergic signaling pathways.

The upregulation of purinergic receptors (P2X4, P2X7, and P2Y2) observed in fatal ZIKV‐associated microcephaly supports the hypothesis that excytotoxicity—mediated by excessive or prolonged extracellular glutamate accumulation—may be a contributing factor [9]. In the context of ZIKV immunopathogenesis, the inflammatory milieu is characterized by Th1, Th17, and M1 microglial activation. Purinergic receptor engagement can enhance glutamate release from glial cells, leading to hyperactivation of glutamatergic receptors, such as the N‐methyl‐d‐aspartate (NMDA) receptor [21]. This, in turn, increases intracellular Ca²⁺ concentrations and promotes ROS and RNS production, driving oxidative stress and cellular injury while compromising BBB permeability [22, 23].

In congenital Zika syndrome, neuroinflammation is further enhanced by inflammasome activation, which promotes the expression of inducible nitric oxide synthase (iNOS) and subsequent nitric oxide production [24]. In this context, purinergic signaling may provide valuable insights into the mechanisms of neuronal death, which is marked by the widespread accumulation of ROS, which is extremely toxic to both resident and infiltrating immune cells [25].

It is worth noting that the investigation was conducted with a limited sample size, consisting of six individuals affected by ZIKV and three controls. This represents a significant limitation for the statistical robustness of the findings. Although the small number of participants may limit the study's power, it was possible to identify statistically significant differences between the analyzed groups.

These results highlight the relevance of the study, as they provide important preliminary data on ZIKV infection and neuroinflammation. Considering the scarcity of available samples for this type of analysis, the findings obtained contribute significantly to the advancement of knowledge in the field, serving as a basis for future investigations with larger samples.

## 5 Conclusions

The immunopathogenesis of fatal ZIKV‐associated microcephaly involves a complex interplay between endothelial activation, leukocyte recruitment, and purinergic signaling, all of which contribute to neuroinflammation, oxidative stress, and neuronal death. The increased expression of adhesion molecules and purinergic receptors highlights the central role of immune‐mediated vascular dysfunction in disrupting BBB integrity. These findings underscore the importance of further investigating the molecular mechanisms linking endothelial signaling to excytotoxicity during ZIKV infection, which may guide the development of targeted therapies to mitigate neurodevelopmental damage in congenital Zika syndrome.

## Author Contributions

J.R.S., J.A.S.Q., and P.F.C.V. were responsible for the conceptualization of the study. G.B.F., L.V.T., C.S., L.L., O.N., J.O.C., L.M.N.C., and R.A. contributed to the development of the methodology. J.O.C., L.L., and A.J.M.F. were involved in data curation. L.C.M., R.A., A.J.M.F., and L.M.N.C. performed the formal data analysis. J.R.S., G.B.F., L.V.T., L.C.M., J.A.S.Q., and P.F.C.V. wrote the original draft of the manuscript. J.R.S., J.A.S.Q., and P.F.C.V. reviewed and edited the manuscript. and J.R.S., J.A.S.Q., A.J.M.F., L.C.M., L.L., and P.F.C.V. supervised the study. All authors read and approved the final version of the manuscript.

## Ethics Statement

Patient samples were collected and processed as part of surveillance activities in response to the ZIKV epidemic in Brazil, conducted on an emergency basis in accordance with guidelines established by the Brazilian Ministry of Health. This study was approved by the Research Ethics Committee (CEP) of the Evandro Chagas Institute (IEC), under approval number 1.888.946. All procedures were carried out in accordance with the standards and guidelines set forth by the CEP/IEC, as well as the regulations of the Brazilian Ministry of Health for studies involving biological samples.

## Consent

The authors have nothing to report.

## Conflicts of Interest

The authors declare no conflicts of interest.

## Data Availability

The authors have nothing to report.
